# Knowledge, perceptions and practices on antimicrobial resistance in humans and animals in Wakiso district, Uganda: A cross sectional study

**DOI:** 10.1371/journal.pgph.0002701

**Published:** 2023-12-13

**Authors:** David Musoke, Grace Biyinzika Lubega, Michael Brown Obeng, Claire Brandish, Jody Winter, Filimin Niyongabo, Kate Russell-Hobbs, Bee Yean Ng, Lawrence Mugisha, Saba Amir, Freddy Eric Kitutu, Linda Gibson

**Affiliations:** 1 Department of Disease Control and Environmental Health, School of Public Health, College of Health Sciences, Makerere University, Kampala, Uganda; 2 Institute of Health and Allied Professions, School of Social Sciences, Nottingham Trent University, Nottingham, United Kingdom; 3 Pharmacy Department, Buckinghamshire Healthcare NHS Trust, Aylesbury, United Kingdom; 4 Department of Biosciences, School of Science and Technology, Nottingham Trent University, Nottingham, United Kingdom; 5 Department of Pharmacy, Oxford University Hospitals NHS Foundation Trust, Oxford, United Kingdom; 6 Department for Livestock and Industrial Resources, School of Veterinary Medicine and Animal Resources, College of Veterinary Medicine, Animal Resources and Biosecurity (COVAB), Makerere University, Kampala, Uganda; 7 School of Animal, Rural and Environmental Sciences, Nottingham Trent University, Nottingham, United Kingdom; 8 Department of Pharmacy, School of Health Sciences, College of Health Sciences, Makerere University, Kampala, Uganda; University of St. Andrews, UNITED KINGDOM

## Abstract

Despite increasing evidence on antimicrobial resistance (AMR), there is limited literature on antimicrobial access and use in humans and animals in community settings globally. This study assessed knowledge and perceptions of AMR, as well as practices relating to the use of antimicrobials in humans and animals in Wakiso district, Uganda. This was a cross-sectional study among 418 participants that employed quantitative data collection methods. A structured questionnaire that included questions on knowledge, perceptions, practices related to AMR, and perceptions on access to antimicrobials in humans and animals was used. Data was analysed in STATA version 10. The majority of participants 63.6% (266/418) had heard about AMR mainly from family and friends 57.5% (153/266), and most 70.8% (296/418) were aware that resistant microorganisms cause infections that are difficult to treat. Most participants 62.7% (262/418) thought that they should complete the full dose of antimicrobials when on treatment. However, on the last occasion of antimicrobial use, 13.0% (44/338) revealed that they did not complete the full course of treatment. Participants who were single (APR = 1.12, C.I = 1.03–1.12, p-value = 0.01) and earning between 91 and 290 USD on average per month (APR = 1.12, C.I = 1.02–1.23, p-value = 0.02) were more likely to have completed a given antimicrobial course as compared to those who were married/cohabiting and earned less than 15 USD respectively. The majority of participants 60% (251/418) owned animals, and 81.3% (204/251) reported using antimicrobials mainly for prevention 61.3% (125/204) or treatment of sick animals 70.6% (144/204). Among the participants, 57.4% (117/204) reported not having sold or consumed animal products within a week after exposure to antimicrobials. Interventions to prevent AMR should adopt a One Health approach to address the gap in knowledge and practices relating to the use of antimicrobials in humans and animals.

## Introduction

In 2019, 1.27 million deaths were estimated to be directly attributable to drug-resistant bacterial infections globally, with sub-Saharan Africa suffering the highest burden [1]. Inappropriate use of antimicrobials has been identified as a leading cause of antimicrobial resistance (AMR) in low- and middle-income countries (LMICs) [2]. According to the World Health Organization (WHO), understanding the current use of antimicrobials in human and animal health is the first step in developing strategies to address barriers to the appropriate use of medicines [3]. The barriers to appropriate use of antimicrobials globally are many, ranging from lack of knowledge about proper use in communities, farms, and health care facilities, to the lack of government policies and political will [4]. In addition, self-medication and premature discontinuation of antimicrobials are evident in both human and animal health particularly in low-income settings. Poor health-seeking habits where patients expect an antimicrobial either for themselves or their animals even when not required have a negative influence on practitioners’ prescribing practices in many countries including Uganda [5, 6]. In the animal sector, antimicrobials are often used inappropriately to maximise production yields and sales profits. Sub-therapeutic doses of antimicrobials are reportedly used in animal feed for increased efficiency and disease prevention in addition to treating infections [7]. In the environment, inappropriate disposal of antimicrobials within household waste, as well as in wastewater drainage channels and water bodies has been reported [8, 9]. Given the interrelatedness of humans, animals and the environment, global frameworks have at their core the use of a One Health approach which calls for integrated action across all sectors to address AMR [2, 3].

In Uganda, at least four in every ten people seeking medical care are prescribed an antibiotic [10]. Previous prevalence levels of antibiotic use range from 39% to 44% [11] in humans, and from 66% to 96% in animals [12, 13]. A recent cross-sectional study among humans and animals found a high frequency (69% in Namuwongo ward, Kampala district and 75% in Nagongera town council, Tororo district) of antibiotic use in households and farms [14]. Due to limited access to health care in Uganda such as only 72% of households living within 5 km of a health facility, individuals are likely to self-medicate with antibiotics [15, 16]. Veterinarians in Uganda have been reported to issue out antibiotics to avoid conflict with farmers who will endure financial impact due to the loss of their livestock [6]. The patterns of antibiotic use in both humans and livestock in Uganda are largely dependent on geographical proximity to health services and economic status of humans [14]. Communities that are distant from health facilities and have a lower income status generally have reduced access to antibiotics and rely on government structures [17]. These challenges all contribute to the increased development of AMR across the country. It is important to understand the rationale behind barriers to appropriate antimicrobial use so that sustainable solutions based on behaviour change principles can be developed. In 2021, WHO endorsed the UN Sustainable Development Cooperation Framework, recognising the importance of tackling AMR to achieve some of the Sustainable Development Goals [2, 18]. In response to this, Uganda developed a 5-year AMR National Action Plan (NAP) in 2018, which sets out a framework of actions to address AMR within the country using a One Health approach [19].

Literature on the trends of antibiotic use in human health care, livestock and aquaculture settings in Uganda is increasing [12, 20–22]. However, there is still limited evidence on antimicrobial access and use in humans and animals with a focus on community settings using the One Health approach [23, 24]. Indeed, vast literature on antimicrobial use in the country is primarily on humans and health facility based particularly hospitals [16, 23]. This study, therefore, assessed knowledge and perceptions on AMR, as well as practices on the use of antimicrobials in humans and animals in Wakiso district, Uganda. The results of the study could be used to raise awareness, as well as empower communities to make informed decisions and change their health-seeking behaviours to promote antimicrobial stewardship (AMS). Such data is key to inform actions for medical, veterinary, environmental, and other professionals as well as policy makers on tackling AMR and strengthening AMS through the One Health approach as stipulated in Uganda’s AMR NAP [19]. Furthermore, the findings from this study contribute to Agenda 2063: *the Africa We Want* which also considers One Health as a sustainable solution to making Africa safer and healthier for humans, animals, plants and their shared environment [25].

## Methods

### Study area and participants

The study was carried out in Kajjansi and Kasanje town councils in Wakiso district, central Uganda. Wakiso’s proximity to the country’s capital, Kampala, allows for easy access to a range of antimicrobials for treatment of infections in both humans and animals. The district consists of four municipalities, seven sub-counties, and eight town councils, and half of its population lives in rural areas [26]. Kajjansi and Kasanje town councils were chosen for the study due to their composition of both urban and rural populations. Kajjansi town council had 23,992 households with a population of 93,238, while Kasanje town council had 7,825 households with a population of 30,276 [26]. Kasanje town council had only one government health facility (Kasanje health centre III) while Kajjansi town council had 3 government health facilities (Kajjansi health centre IV, Nakawaka health centre III, and Nsaggu health centre II). Both town councils have several private clinics, pharmacies, and drug shops which are all involved in dispensing and prescribing antimicrobials [27]. The main economic activities in the area include subsistence agriculture, brick making, small-scale businesses, and animal husbandry [28]. The study participants were household members above 18 years, with priority given to household heads or other responsible adults such as their spouses. These household heads and other adults were expected to generally be knowledgeable on access and use of antimicrobials and other issues related to AMR in their respective households. Only one participant per household was involved in the study.

### Study design and data collection

This was a cross-sectional study that employed quantitative data collection methods. A minimum sample of 385 was calculated using the formula by Kish and Leslie, with a 95% confidence interval, precision of 5% and Z value of 1.96 [29]. A list of all parishes in the two town councils (11 in Kajjansi and 7 in Kasanje) was obtained from the Wakiso district health office. Two parishes were randomly selected from each town council to be involved in the study. From the two parishes, a list of villages was provided by the district planning office from which four villages from each parish were randomly selected. Sampling proportionate to size was used to determine the number of households to participate in the study from each village. A health facility or local council office was considered as the point of reference to select the first household to participate in the study. Thereafter, a sampling interval of five was used. The questionnaire was administered for approximately 40 minutes by 8 research assistants who had bachelors degrees in science-related courses including pharmacy, medicine, and environmental health.

All the research assistants were conversant in *Luganda* which is the most commonly used local language in Wakiso district. The research assistants were trained by the researchers for two days on how to administer the tool correctly while observing objectivity, ethics, and professional conduct in the field. After the training of research assistants, the questionnaire was piloted in another town council that was not part of the study and revised accordingly prior to data collection. This piloting enabled the research team to revise the tool in order to ensure that the questions were clear and captured the desired information. The questionnaire was developed in reference to various literature on the use and access of antimicrobials in relation to One Health [14, 30]. The questionnaire comprised of the following sections: socio-demographic characteristics of participants (age, sex, education, income, and occupation); knowledge, perceptions and practices on AMR and related factors (impact, causes, and prevention of AMR such as handwashing); perceptions on access to antimicrobials in humans (source of antimicrobials, safety, affordability, accessibility and effectiveness of antimicrobials); as well as ownership and antimicrobial practices among animals (sources, uses and disposal of antimicrobials, and withdrawal periods). Specifically, practices related to AMR included those on access, disposal and seeking advice on antimicrobials.

### Data management and analysis

All completed questionnaires were checked for completeness by two field supervisors at the end of each data collection day. Missing data was handled by checking with the respective research assistants and in some instances getting clarification from the field. After collection, data (S1 Data) was entered into EPIDATA version 3.0, and then transferred to STATA version 10 for data cleaning and analysis. Descriptively, participant responses were analysed and presented as frequencies and percentages for categorical variables, and as mean and standard deviation for continuous variables. Multivariate analysis using modified Poisson regression was used to investigate the association between completion of a given antimicrobial course and the independent variables. A p-value of <0.05 was considered to be statistically significant.

### Ethical considerations

Ethical approval to conduct the study was granted by the Makerere University College of Health Sciences, School of Health Sciences Research and Ethics Committee (2019–051), and the Uganda National Council for Science and Technology (HS 2711). Permission to collect data was obtained from the Wakiso District Health Office and the Local Council 1 leaders of the selected villages. The purpose of the study including benefits and potential risks was explained to the participants in simple, clear terms, and written consent was obtained prior to the start of data collection. Confidentiality and anonymity were ensured, and data from the study was solely used for research purposes.

## Results

### Socio-demographic characteristics of participants

A total of 418 participants from eight villages participated in the study including 75.6% (316/418) females and 35.4% (148/418) aged between 18–29 years, with a mean age of 40 years (SD 16.4). Among the participants, 62.2% (260/418) were married / co-habiting, and 49.3% (206/418) had attained primary education. The majority of participants were self-employed 66.3% (277/418), and 59.1% (247/418) had an average monthly income of less than 15 US dollars (Table 1).

**Table 1:** Socio-demographic characteristics of participants.

Variable	Frequency (n = 418)	Percentage (%)
**Age (years)**		
**Mean age (standard deviation)**	40.1 (16.4)	
18–29	148	35.4
30–39	86	20.6
40–49	72	17.2
50–59	43	10.3
≥ 60	69	16.5
**Sex**		
Female	316	75.6
Male	102	24.4
**Marital status**		
Married / cohabiting	260	62.2
Divorced / separated	55	13.2
Widowed	44	10.5
Single	59	14.1
**Highest level of education**		
None	71	17.0
Primary	206	49.3
Secondary (ordinary)	103	24.6
Secondary (advanced)	12	2.9
University / tertiary	26	6.2
**Occupation**		
Unemployed	55	13.2
Self-employed	277	66.2
Employed by organisation	44	10.5
Housewife	42	10.1
**Average income per month (USD)**		
< 15	247	59.1
15–90	130	31.1
91–290	41	9.8
**Household position**		
Household head	193	46.2
Spouse of household head	196	46.9
Relative of household head	29	6.9

### Knowledge and perceptions on antimicrobial resistance

Among the participants, 63.6% (266/418) had heard about AMR, mainly from family and friends 57.5% (153/266) and health professionals 30.1% (80/266). The majority of participants 70.8% (296/418) were aware that resistant microorganisms cause infections that are difficult to treat. However, 37.1% (155/418) of the participants mentioned that AMR was only a problem for people who took antimicrobials regularly. Almost all the participants 96.7% (404/418) agreed that people should use antimicrobials only when prescribed by a health professional. Furthermore, 91.1% (383/418) of the participants agreed that health practitioners should only prescribe antimicrobials when needed (Table 2).

**Table 2:** Knowledge and perceptions on antimicrobial resistance.

**Variable**	**False**		**True**		**Did not know**	
	**n = 418**	**%**	**n = 418**	**%**	**n = 418**	**%**
If microorganisms are resistant to antimicrobials, it can be very difficult to treat the infections they cause	63	15.1	296	70.8	59	14.1
Antimicrobial resistance is an issue that could affect me or my family	25	6	347	83	46	11
Antimicrobial resistance is an issue in other countries / places but not in my community	239	57.2	101	24.2	78	18.7
Antimicrobial resistance is a problem for only people who take antimicrobials regularly	181	43.3	155	37.1	82	19.6
Disease causing microorganisms which are resistant to antimicrobials can be spread from person to person	83	19.4	254	60.8	81	19.4
	**Agreed**		**Disagreed**		**Did not know**	
	**n = 418**	**%**	**n = 418**	**%**	**n = 418**	**%**
People should use antimicrobials only when they are prescribed by a health professional	404	96.7	11	2.6	3	0.7
Farmers should use antimicrobials sparingly among food-producing animals	288	68.9	39	9.3	91	21.8
People should not keep antimicrobials and use them later for other illnesses	343	82.1	64	15.3	11	2.6
Parents should make sure all their children’s vaccinations are up-to-date	410	98.1	4	1	4	1.0
People should wash their hands regularly	418	100				
Health professionals should only prescribe antimicrobials when they are needed	383	91.6	4	1	31	7.4

Most of the participants 62.7% (262/418) thought that they should finish the full antimicrobial dose once on treatment. With reference to the last occasion when antimicrobials had been taken, the majority of participants 90.9% (307/338) reported having received a prescription, and 95.6% (323/338) had been advised on how to take the antimicrobials by a health professional. Among the 338 participants who acquired antimicrobials on the last occasion of their sickness, 13% (44/338) did not complete the full course of treatment. The main reason given for this practice was that they felt better and no longer had any symptoms 72.7% (32/44).

### Factors associated with the completion of a given antimicrobial course

Using modified Poisson regression analysis, participants who were single (APR = 1.12, C.I = 1.03–1.12, p-value = 0.01) and earning more than 90 USD on average per month (APR = 1.12, C.I = 1.02–1.23, p-value = 0.02) were more likely to have completed a given antimicrobial course compared to those who were married / co-habiting and earning less than 15 USD on average per month respectively. Participants who had antibiotics at home (APR = 0.86, C.I = 0.75–0.10, p-value = 0.05) were less likely to have completed the given antimicrobial course compared to those who did not have antibiotics at home (Table 3).

**Table 3:** Factors associated with the completion of a given antimicrobial course.

Independent variables	Completion of a given antimicrobial course			
	Crude Prevalence Ratios (CPRs) at 95% CI	p-value	Adjusted Prevalence Ratios (APRs) at 95% CI	p-value
**Age (years)**				
18–26	1		1	
27–33	1.10 (0.98–1.23)	0.09	1.11(1.00–1.25)	0.04
34–48	0.98 (0.86–1.12)	0.82	0.99 (0.87–1.12)	0.90
49–100	1.06 (0.96–1.21)	0.21	1.03 (0.92–1.16)	0.57
**Marital status**				
Married / cohabiting	1		1	
Single	1.10 (1.02–1.19)	**0.01**	1.12 (1.03–1.22)	**0.01**
**Average income per month (USD)**				
< 15	1		1	
16–90	0.97 (0.88–1.07)	0.52	0.96 (0.88–1.06)	0.43
> 90	1.11 (1.02–1.21)	**0.02**	1.12 (1.02–1.23)	**0.02**
**Got advice from a health worker**				
No	1		1	
Yes	1.19 (0.88–1.63)	0.26	1.22 (0.99–1.50)	0.06
**Had an antimicrobial at home**				
No	1		1	
Yes	0.85 (0.73–0.89)	**0.03**	0.86 (0.75–0.10)	**0.05**

### Perceptions on access to antimicrobials for use in humans

The majority of participants 87.1% (364/418) agreed that private pharmacies / drug shops nearest to their households usually had the antimicrobials they needed. Nearly half 49.0% (205/418) of the participants agreed that their nearest government health facility usually had the antimicrobials they needed. Most participants 94.7% (396/418) agreed that antimicrobials were more expensive at private pharmacies than at public healthcare facilities. The majority of participants 71.8% (300/418) agreed that their households could usually afford to buy the antimicrobials they needed. However, 45.2% (189/418) of participants reported of the need to have borrowed money or sold household items in order to pay for antimicrobials in the past. Regarding the quality of services, 81.4% (340/418) of the participants agreed that private healthcare providers in their neighbourhood were good compared to 54.9% (229/418) for government health facilities (Table 4).

**Table 4:** Perceptions on access to antimicrobials for use in humans.

Variable	Agreed		Disagreed		Did not know	
	n = 418	%	n = 418	%	n = 418	%
The government health facility nearest to my household is easy to reach	272	65.1	140	33.5	6	1.4
My household would use government health facilities more if opening hours were convenient	308	73.7	83	19.9	27	6.4
The government health facility nearest to my household usually has the antimicrobials we need	205	49.0	170	40.7	43	10.3
The private pharmacy / drug shop nearest to my household usually has the antimicrobials we need	364	87.1	28	6.7	26	6.2
My household can get free antimicrobials at the government health facility	336	80.4	52	12.5	30	7.2
Antimicrobials are more expensive at private pharmacies than at public health care facilities	396	94.7	8	1.9	14	3.4
My household can usually get antimicrobials on credit from the private pharmacy / drug shop if needed	195	46.7	214	51.2	9	2.2
My household can usually afford to buy the antimicrobials we need	300	71.8	108	25.8	10	2.4
In the past, my household had to borrow money or sell items to pay for antimicrobials	189	45.2	219	52.4	10	2.4
The quality of services delivered at government health facilities in my neighbourhood is good	229	54.8	144	34.5	45	10.8
The quality of services delivered by private health care providers in my neighbourhood is good	340	81.4	39	9.3	39	9.3

### Ownership and practices relating to antimicrobial use in animals

The majority of participants 60% (251/418) reported having animals in their households. The commonly owned animals were pigs 57.0% (143/251), poultry 49.8% (125/251), goats / sheep 35.9% (90/251), cattle 32.3% (81/251), dogs / cats 4.8% (12/418), and rabbits 1.2% (3/418). Of the 251 participants who owned animals, the majority 81.3% (204/251) reported using antimicrobials among them. Most participants 70.6% (144/204) reported using antimicrobials to treat sick animals, 61.3% (125/204) to prevent animals from falling sick, while 13.7% (28/204) for growth promotion. However, 37.1% (93/251) of the participants did not know the type of antimicrobials that were being administered to treat their animals. Most participants 74.5% (152/204) stated that veterinary workers were the main source of antimicrobials and administered them to their animals. Among the 72.1% (147/204) participants who normally sought advice about their animals, the majority 91.8% (135/147) received this from veterinary workers. Of the 57.4% (117/204) participants who reported not having sold or consumed animal products within a week after medication, 46.2% (54/117) sold or consumed their animal products after a specified time as recommended by veterinary workers. In addition, most participants disposed of unwanted antimicrobials, sachets and bottles through burning 34.3% (70/204), throwing them into pit latrines, rubbish pits, or drains / open areas 38.7% (79/204), while only 18.6% (38/204) returned unused antimicrobials to veterinary workers for disposal (Table 5).

**Table 5:** Practices relating to antimicrobial use in animals.

	Frequency	Percentage (%)
**Common reasons for administering antimicrobials to animals** *	**n = 204**	
To treat sick animals	144	70.6
To prevent animals from becoming sick	125	61.3
To fatten / increase growth of the animals	28	13.7
Others	5	2.5
**Categories of animals commonly administered with antimicrobials** *	**n = 204**	
Only sick animals	77	37.8
All animals of the same species	101	49.5
All animals including of different species	18	8.8
Others	4	2.0
**Commonly used sources of antimicrobials** *	**n = 204**	
Veterinary worker	152	74.5
Veterinary drug shop	39	19.1
Human medicine pharmacy / drug shop	7	3.4
Others	9	4.4
**Households which sought advice on use of antimicrobials in animals before use**	**n = 204**	
Yes	147	72.1
No	57	27.9
**Commonly used avenues for getting advice on use of antimicrobials in animals**	**n = 147**	
Veterinary worker	135	91.8
Human health professionals	1	0.7
Other farmers	13	8.2
Package label	1	0.7
Drug shop attendant	3	2.7
**Common duration of usage of antimicrobials** *	**n = 204**	
As recommended by a veterinary worker or other providers	152	74.5
Until the animal is cured	29	14.2
Until the package is empty	5	2.5
Once	7	3.4
As long as they could afford	1	0.5
Others	17	8.3
**Commonly used administrator of antimicrobials to animals**	**n = 204**	
Member of the household	21	10.29
Owner of the animal	27	13.24
Veterinary worker	151	74.02
Animal attendant	5	2.45
**Commonly used route for administration of antimicrobials to animals** *	**n = 204**	
Oral	101	49.5
Injection	159	77.9
With water	49	24.0
With feed	11	5.4
With spray	3	1.5
**Sold or consumed animal products (milk, meat or eggs) from animals that were treated with antimicrobials within 7 days**	**n = 204**	
Yes	87	42.7
No	117	57.4
**Basis for duration taken between treatment of animals and consumption / selling of their product**	**n = 117**	
Manufacturer’s recommendation	7	6.0
As per advice of a veterinary worker	54	46.2
Own judgement	45	38.5
Others	11	9.4
**Common disposal methods for expired animal antimicrobials, empty bottles and sachets** *	**n = 204**	
Collected from residence by solid waste collection entities	7	3.4
Deposited in communal bins and collected by solid waste collection entities	7	3.4
Placed in a rubbish pit next to house	16	7.8
Placed in a communal rubbish pit	9	4.4
Burned	70	34.3
Thrown in drain / open area	11	5.4
Thrown in pit latrine	43	21.1
Collected by veterinary worker	38	18.6
Others	34	16.7

* Multiple choice responses

## Discussion

This study presents findings on knowledge and perceptions of AMR, as well as practices relating to the use of antimicrobials in humans and animals in a community setting in Wakiso district, Uganda. Despite participants’ good knowledge and perceptions of certain aspects of AMR such as the use of antimicrobials only when prescribed by health professionals, practices on use of antimicrobials in both humans and animals were generally poor. For example, not completing the full course of treatment in humans, poor disposal of expired animal antimicrobials, and using own judgement as a basis for determining the duration between treatment of animals and consumption / selling of their products were established in the study. Our findings offer an understanding of the complexities of antimicrobial use in community settings and present some of the barriers to putting knowledge into action to enhance AMS. This study provides a spotlight on both human and animal health thereby contributing to data on AMR in community settings, an area that is still understudied and misunderstood [23, 31]. Therefore, the study provides evidence of the need for sustainable solutions that consider all aspects of behaviour change to help tackle AMR by promoting a holistic view of AMS among humans and animals using a One Health approach.

Despite many participants in our study earning a meagre average monthly salary of less than 15 USD and (45.2%) of them reported to having sold items or borrowed money in the past in order to access antimicrobials, the majority (71.8%) considered antimicrobials accessible and usually affordable. Communities in districts such as Wakiso that have many private and public health facilities are likely to have greater access to antimicrobials [27]. In our study, participants who were higher income earners were more likely to have completed a given antimicrobial course. Economic status can influence the pattern of antimicrobial use in humans [17]. Indeed, a study on acute respiratory infections (RTIs) in Uganda also reported increased antibiotic usage among households with higher income status [11]. Another possible reason is that higher earners are less likely to worry about the affordability of drugs in the future [16]. High-income earners may also have attained a higher level of education hence literacy that would promote their adherence to public health messaging on medication. On the contrary, people with very low income are always under financial pressure and therefore might not understandably prioritise purchasing full doses of medicine [32], despite understanding the importance of completing the treatment course as stipulated by (67%) of the participants. Evidence on shorter courses of antibiotics, particularly for RTIs favouring lesser days is promising for completeness of dosage [33]. Shorter course length for antimicrobials could therefore be considered whenever feasible to enhance patients completing of the dosage.

Participants who were single were more likely to have completed a given antimicrobial course. This may be because individuals who are single usually stay alone and may have less reason to hold back part of a course of antimicrobials for use by others, in cases where the infection is transmitted to several members of a household. This is in line with the finding from our study that those who had an antimicrobial at home were less likely to complete a given antimicrobial course. This could suggest that, although a full antimicrobial course is prescribed, the recipient might reserve a portion of it at home for future use by themselves or family members [34–36]. Indeed, (72.7%) of participants in our study reported that the main reason for not completing the whole antimicrobial course was the resolution of the symptoms. This finding is similar to studies done in India [37] and Uganda [16] which found that resolution of signs and symptoms was associated with not completing an antimicrobial dose. More education and awareness are needed on the appropriate use of antimicrobials specifically from health professionals to ensure the optimal and intended effects of the use of these medicines.

Participants indicated that antimicrobials from private pharmacies and drug shops nearest to their households were expensive. High healthcare costs especially out-of-pocket expenditure is a major contributor to poor health-seeking behaviours [7, 38, 39]. This could also potentially deter community members from seeking health information from health professionals therefore increasing self-medication practices [14]. Participants indicated that their main source of information about AMR (57.5%) was from their families, friends, and close relatives rather than health professionals. Another study also asserted that a vast proportion of the population in community settings did not seek health information from health professionals [16]. This is supported by the Ministry of Health (Uganda) report which stated that the lack of health information in most cases causes people to self-medicate [15]. In our study, participants who had received advice from a health worker were more likely to complete the course on antimicrobials. This suggests that interventions aimed at promoting AMS should be community centred, with more emphasis on increasing awareness on AMR and its implications on cost-effective treatments in future.

Participants in our study had relatively good knowledge on AMR and how antimicrobials should be used in humans, with a few misconceptions evident. Majority (49.3%) of participants in our study had at least primary education, which could have influenced their knowledge of the appropriate use of antimicrobials. Indeed, the majority of participants were aware that resistant infections may be difficult to treat and could cause problems for them or their families. In addition, many participants agreed that antimicrobials should be used only when prescribed by a health professional. General knowledge about the importance of infection prevention measures such as hand washing (100%), and childhood vaccinations (98.1%) were also high in our study. This is different to previous reports which showed lack of knowledge about the appropriate use of antimicrobials [4, 24, 39]. Contrarily to our study, a recent study in Kasese, Uganda found that 78% of surveyed pastoralists did not know about AMR [40]. This result differs from our study probably because it was carried out in the Western region of the country. It is also possible that earlier interventions in Wakiso district such as the training of community health workers on AMS [30] may have increased community knowledge.

Among participants who kept animals, pigs and poultry were the most prevalent. This finding is similar to other studies in Kampala [41] and Wakiso [12] districts which found that piggery and poultry farming where highly practiced. This finding is not surprising as Uganda’s main economic activity is agriculture including animal husbandry particularly in rural areas [42, 43]. Indeed, it is common for households to have animals for food security and sale in Uganda [42–44]. In our study, few (13.7%) participants reported using antimicrobials to fatten or increase the growth of their animals. This is encouraging and suggests a change in knowledge and resulting behaviours when compared to a previous study conducted earlier which reported 90% of households using antimicrobials as growth promotors [45]. However, prophylactic administration of antimicrobials to animals that were not sick (61.3%) was common in our study. This finding is similar to previous studies where households were using antimicrobials for disease prevention in Uganda [45], Kenya [46] and five other African countries [47]. This calls for interventions to raise awareness on measures to prevent diseases in animals as opposed to relying on antimicrobials. This could be a powerful driver to reduce inappropriate use of antimicrobials in animals among communities [6].

The majority of participants in our study reported obtaining antimicrobials for use in animals from veterinary workers (74.5%) and using them for the duration recommended by the provider (74.5%). In addition, (91.8%) reported that they commonly received advice on the use of antimicrobials in animals from veterinary workers. Similar findings regarding advice on the use of antimicrobials were found in Bangladesh commercial chicken production farmers [48] and pig farmers in Uganda [49]. This finding is different from other studies where advisory input from animal health professionals was rare [45, 47]. More research is needed to ascertain the facilitators of performance among veterinary officers in animal welfare. In addition, since many community members rely on the advice of veterinary workers, AMS efforts should focus on supporting veterinary professionals to promote appropriate alternatives to using antimicrobials for veterinary prophylaxis and growth promotion. This would minimize the inappropriate use of antimicrobials as a result of unqualified and unknowledgeable veterinary officers [45, 48, 50]. Most of the participants in our study who owned animals disposed unwanted antimicrobials, sachets and bottles through burning, throwing them into pit latrines, rubbish pits and open drains, yet inappropriate disposal in the environment is an important driver of AMR. In England, antibiotic amnesty campaigns are being conducted to raise awareness on risks of poor antimicrobial disposal. As part of this campaign, people are encouraged to return of expired and unwanted antibiotics to community pharmacies for safe disposal [51]. We recommend use of a similar campaign in Uganda with a focus on proper disposal of expired and unused animal antimicrobials, and emphasis on raising awareness on the role of the environment in AMR.

Our study took a One Health approach which is a strength particularly in this era when working across disciplines is being encouraged. Another strength of the study is that it focused on knowledge, perceptions and practices relating to antimicrobial use in communities, while the majority of previous studies relating to AMS in Uganda have focused on larger healthcare facilities [52, 53]. However, this study relied on self-reported responses from participants hence the possibility of recall bias, especially for questions that required participants to remember past events. Another limitation is that the study was only carried out in two town councils within Wakiso district, hence the findings may not be applicable to other settings.

## Conclusion

Participants were aware of many aspects of AMR and had good perceptions on the need to take steps to avoid infections and seek advice from human and animal health professionals before taking antimicrobials or giving them to animals. However, inappropriate practices such as not completing the prescribed antimicrobial course and using antimicrobials to prevent animals from becoming sick were commonly encountered. Future interventions tackling AMR should take a One Health approach to help address the gaps in knowledge and sub-optimal practice relating to antimicrobial use in humans and animals at community level.

## Supporting information

S1 Data Dataset. (DTA)
